# Age-Related Changes in Skeletal Muscle Oxygen Utilization

**DOI:** 10.3390/jfmk7040087

**Published:** 2022-10-14

**Authors:** Sabrina S. Salvatore, Kyle N. Zelenski, Ryan K. Perkins

**Affiliations:** Department of Kinesiology, California State University Chico, 400 W 1st St, Chico, CA 95929, USA

**Keywords:** aging, muscle oxygen saturation (SmO_2_), near-infrared resonance spectroscopy (NIRS), muscle metabolism

## Abstract

The cardiovascular and skeletal muscle systems are intrinsically interconnected, sharing the goal of delivering oxygen to metabolically active tissue. Deficiencies within those systems that affect oxygen delivery to working tissues are a hallmark of advancing age. Oxygen delivery and utilization are reflected as muscle oxygen saturation (SmO_2_) and are assessed using near-infrared resonance spectroscopy (NIRS). SmO_2_ has been observed to be reduced by ~38% at rest, ~24% during submaximal exercise, and ~59% during maximal exercise with aging (>65 y). Furthermore, aging prolongs restoration of SmO_2_ back to baseline by >50% after intense exercise. Regulatory factors that contribute to reduced SmO_2_ with age include blood flow, capillarization, endothelial cells, nitric oxide, and mitochondrial function. These mechanisms are governed by reactive oxygen species (ROS) at the cellular level. However, mishandling of ROS with age ultimately leads to alterations in structure and function of the regulatory factors tasked with maintaining SmO_2_. The purpose of this review is to provide an update on the current state of the literature regarding age-related effects in SmO_2_. Furthermore, we attempt to bridge the gap between SmO_2_ and associated underlying mechanisms affected by aging.

## 1. Introduction

The population of older adults is expanding due to greater life expectancy by means of advancements in medical technology and a more comprehensive understanding of physiological processes. By the year 2030, there is an expected 44% increase in the population of individuals aged 65 and older [1]. However, length of life increases the susceptibility for age-related diseases. As a result, individuals aged ~75 y and older contribute to about two-thirds of the annual 868,662 cardiovascular disease-related deaths [2,3]. Currently, the financial burden associated with cardiovascular disease is USD 555 billion annually and is expected to increase to USD 1.1 trillion by 2035 [4]. Beyond the influence of cardiovascular disease in clinical health, a robust cardiovascular system is also critical to aerobic fitness and exercise performance. More specifically, the cardiovascular system is essential for the delivery of oxygen to meet the metabolic demand of tissues. This requirement is increased during aerobic activity, as working muscles can use as much as 85% of the oxygen that is delivered [5,6,7]. With advancing age, the ability to meet increased metabolic demand gradually becomes more difficult due to the progressive decline in aerobic fitness. The rate of decline in aerobic fitness can be as much as ~1% per year following 30 years of age [8].

Working muscles require a greater supply of oxygen to withstand increased metabolic demand. Oxygen levels are measured within skeletal muscle tissue by determining the muscle oxygen saturation (SmO_2_), depicting the balance between oxygen delivery and consumption [9]. Oxyhemoglobin (O_2_Hb) and deoxyhemoglobin (HhB) are each assessed to calculate total hemoglobin (O_2_Hb + HhB = ThB) and are typically expressed as a percentage (([O_2_Hb/ThB]) × 100) = %SmO_2_ [10,11]. Measurements of SmO_2_ are generally obtained via near-infrared resonance spectroscopy (NIRS) [10], which has been modified from its original use to assess local SmO_2_ and blood flow in a muscle of interest at rest, during exercise, and into recovery. Through the use of this device, insight into the overall age-related reduction in oxygen delivery, utilization, and extraction by skeletal muscle can be gained [12,13,14].

Coinciding with the decline in whole-body oxygen utilization with age (i.e., VO_2max_), studies leveraging NIRS technology have shown similar age-related changes in local skeletal muscle oxygen utilization. Along the continuum from rest to maximal exercise and into recovery, it has been identified that aging reduces local oxygen availability. As age progresses, a variety of factors likely contribute to impairments in the oxygen delivery and utilization cascade, resulting in reductions in SmO_2_ at rest [13,15,16] during aerobic exercise [13,15,16,17,18,19], and prolonging restoration of metabolic homeostasis following exercise [12,15,20,21]. Impaired SmO_2_ may be attributed to lower muscle mass [22,23], insufficient blood flow [24,25], decreased capillary supply and function [26], dysfunctional endothelial cells [27,28], decreased nitric oxide production [28,29], and decreased mitochondrial content and function [30,31,32]. Although there are many potential factors that decrease local SmO_2_, mismanagement of reactive oxygen species (ROS) is suspected to be a major contributor to each of those components within the oxygen delivery cascade. More specifically, the overproduction and insufficient scavenging of ROS are underlying catalysts for decreases in whole-system function, which is exacerbated with advancing age [33]. Therefore, it is likely that damage observed at the level of the tissue is a primary effect of aging and a consequence of excess ROS mismanagement.

Cardiovascular and skeletal muscle function are intrinsically interconnected due to the interface at the cellular level. Together, these systems form a complex network that relies on effective communication to manage oxygen delivery, extraction, and utilization in skeletal muscle. Multiple reports over the last several years have identified age-related alterations in muscle oxygen utilization that serve as potential explanations for reduced exercise capacity with advanced age. Given that these metabolic processes are critical to maintain optimal health and exercise performance, the purpose of this paper is to synthesize and provide an update on the current state of the literature regarding the effects of aging in apparently healthy individuals and muscle oxygen utilization. The goal of this work is to provide a platform that may guide future investigation on the effects of aging on skeletal muscle health (i.e., tailored exercise interventions to enhance SmO_2_).

## 2. Common Methods of Assessment

### 2.1. Historical Perspective

The electromagnetic spectrum was initially used to evaluate oxygenation status of hemoglobin (Hb) by German physiologist Karl von Vierordt in 1876. He observed spectral light changes, by way of the naked eye, to monitor Hb in trans-illuminated human fingers and compared his findings to solutions containing Hb [34,35]. Half a century later, spectroscopy devices were developed and applied in studies that used visible light (400–650 nm) to assess the oxygenation status of Hb in vitro [36,37]. Frans F. Jöbsis of Duke University is credited with initiating the use of NIRS in medical applications in 1977 [38]. Jöbsis discovered a window of light (i.e., 700 nm–1300 nm) that penetrated deeper into living tissue that allowed for the evaluation of organs [38,39]. It was identified that Hb signals provided insight into the oxygen content at the level of the tissue, enabling the ability to monitor the relationship between oxygen delivery and uptake [40]. Following that breakthrough, Britton Chance developed a portable NIRS device in the 1990s to noninvasively and continually monitor muscle oxidative metabolism during exercise [41] by assessing the gradient between oxygen supply and oxygen consumption in vivo (i.e., SmO_2_) [14].

**Figure 1:**
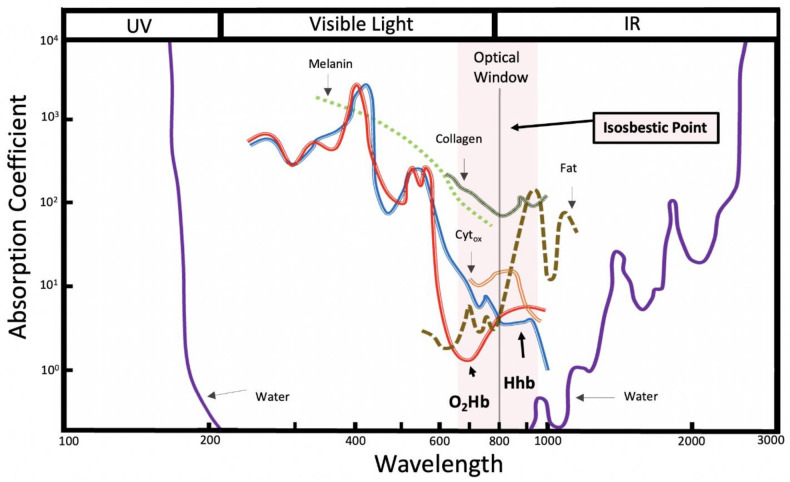
Chromophores present in human tissue plotted against the absorption spectra (natural logarithm base). Water, fat, collagen, deoxygenated hemoglobin (Hhb), oxygenated hemoglobin (O_2_Hb), melanin, and cytochrome oxidase (Cyt_ox_) are observed in the 100 to 3000 nm region. The isosbestic point, the point at which the extinction coefficient of oxygenated and deoxygenated hemoglobin is equal, is plotted at 800 nm. Hhb and O_2_Hb are highlighted as being the primary sources of obtained NIRS signals around the isosbestic point. Y-axis absorption coefficient units: μa [cm^−1^]; X-axis wavelength units: λ [nm]. Reprinted with permission from ref. [45]. Copyright 2014 Elsevier.

The optimal range of light wavelength in NIRS devices used today is ~700–900 nm (Figure 1). This range in wavelength allows for a more effective penetration of biological tissue without interference from water (>900 nm) and scattering of light that occurs in the visible light spectrum (400–700 nm) [34,38,42]. Intravascular Hb, intramuscular myoglobin (Mb), and mitochondrial cytochrome c oxidase (Cyt_ox_) are the primary compounds responsible for the absorption of light in this wavelength region [11,42]. In skeletal muscle, the amount of light absorbed by Cyt_ox_ is suspected to be ≤5% [43,44], suggesting Hb and Mb are the primary sources of obtained NIRS signals [11].

### 2.2. NIRS Validation, Advantages, and Limitations

Briefly, a NIRS device consists of a light source (emitting optode) that emits light at a specified intensity (i.e., the rate at which the energy from the light received is inversely proportional to wavelength), consisting of at least two wavelengths around the isosbestic point, and a light detector (receiving optode) that detects the intensity of the outgoing light. The isosbestic point of the NIRS signal occurs at 800 nm and is the point where the extinction coefficient of oxygenated and deoxygenated hemoglobin is equal [46]. By employing wavelengths around the isosbestic point, a measurement that is more sensitive to oxygenated hemoglobin (800–1000 nm) can be obtained as well as one that is more sensitive to deoxygenated hemoglobin (700–800 nm). The change in light intensity is then translated into clinically valuable information (i.e., SmO_2_ %) [47]. Currently, there are three main classifications for NIRS devices: frequency domain (FD), time domain (TD), and continuous-wave (CW) spectrometers [47] (Table 1). Continuous-wave NIRS devices are the most widely utilized of the devices due to the ease of use and low cost of the device, while the frequency domain and time domain devices are used less frequently due to the requirement for more extensive technological training and cost of the devices [48].

It is important to note the various limitations of NIRS technology that have been reported. Biological tissues are highly scattering, which affects light path length, light absorption, and loss of light penetration due to it being reflected. Regardless of the utilized NIRS device, the thickness of tissue over the muscle of interest, the concentration of melanin in the skin, and adipose tissue thickness (ATT) are factors that affect the signal strength [11]. In addition to those factors affecting signal strength, it has been indicated that collagen is weakly absorbed in the wavelength window that NIRS devices utilize [45]. The path length of light is altered by these variables, but it is also known that variances in signal detection occur with differences in blood volume and motion artifacts [11]. Interestingly, light exposure in the near-infrared spectrum, in addition to visible and ultraviolet light, has been shown to produce excess free radicals in human tissue [49]. This suggests ROS measurements should be interpreted with caution when coupled with NIRS or other light exposure. Nevertheless, NIRS has been validated against other accepted methodologies (i.e., ^1^H-MRS, ^31^P-MRS venous occlusion strain gauge plethysmography, and blood perfusion monitors) showing agreeable signals are obtained regardless of changes in body temperature, skin temperatures, and blood flow [50]. For a more exhaustive review of general NIRS applications to skeletal muscle research, see work by Barstow [11].

**Table 1:** Commonly utilized NIRS devices with associated advantages and limitations.

NIRS Device and Description	Advantages		Limitations
NIR_cw_—Continuous Wave Single Wave Multi-Distance First used for in vivo: ~1977 First used in evaluation of exercise: ~1992 The oldest and most widely used commercial NIRS equipment is the continuous wave (CW) sensor. These devices use a photomultiplier, photodiode, or avalanche photodiode detector to measure light attenuation.	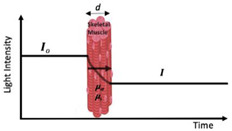	Economical cost Lightweight and portable Sampling rate (i.e., number of readings taken per second) Simplicity and ease of use (i.e., more applicable for monitoring)	Difficult to separate absorption and scattering Limited to monitoring oxygenation trends; however, it is possible to quantify changes in concentrations of chromophores Penetration depth
NIR_TD_—Time Domain (Time-of-Flight or Time-Resolved) First used for in vivo: ~1987 First used in evaluation of exercise: ~2004 Ultrashort pulses typically generated using a semiconductor or solid-state laser. Synchro scan streak camera or a time-correlated single-photon counting method is used to measure photons according to their arrival time.	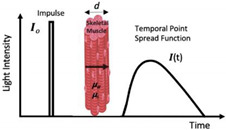	Most accurate spectrometer in separating absorption and scattering. Penetration depth Superior spatial resolution	Cooling required Cost Excessive weight and size. Lack of stabilization. Sampling rate (i.e., number of readings taken per second)
NIR_FD_—Frequency Domain (Frequency-Resolved or Intensity/Phase Modulated Systems) First used for in vivo: ~1995 First used in evaluation of exercise: ~1995 Photon-counting detector or a gain-modulated area detector is employed to assess the attenuation, phase shift (Φ), and modulation (M) depth of the outgoing light.	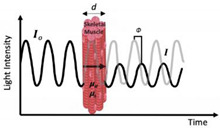	Relative accuracy in uncoupling of absorption and scattering effects Sampling rate (i.e., number of readings)	Cost Complexity of use Excessive size of device Lack of scalability Penetration depth Radio frequency-modulated light cannot exceed 200 MHz Note: The above limitations have slowed NIRS_FD_ translation to clinical applications.

*d*: thickness of the medium; *I*_0_: incident light signal; *I*: transmitted light signal; φ: phase delay; *μa*: 146 absorption coefficients; *μs*: scattering coefficient; *φ*: phase delay; I(t): temporal point spread function of the transmitted light signal. Figures within table are reprinted with permission from ref. [45]. Copyright 2014 Elsevier. For more information, see references [47,51,52,53,54,55,56,57,58,59,60].

## 3. Aging and Muscle Oxygen Utilization

The cardiovascular and skeletal muscle systems, although distinct in their specific system tasks, share the joint responsibility of meeting metabolic demand. To accomplish this, these systems cooperate to supply adequate oxygen to working muscles. Overall, the assessment of oxygen supply and utilization at the level of the tissue (i.e., SmO_2_) has revealed an apparent primary effect of aging. In fact, healthy aging (>65 y) reduces SmO_2_ at rest (~38%), during submaximal (~24%) and maximal (~59%) exercise [13,15]. In addition, the time required for SmO_2_ restoration following exercise in older adults dramatically exceeds that of their younger counterparts [13,15]. It is essential that the cardiovascular and skeletal muscle systems respond quickly to meet changes in metabolic demand. However, the inability to meet current demand may indicate that one or more components involved in the oxygen delivery cascade are impaired.

### 3.1. Submaximal Exercise

In general, there is an inverse relationship between SmO_2_ and exercise intensity in healthy individuals, both young and older. More specifically, SmO_2_ decreases in response to increasing exercise intensity. However, this effect appears to be more pronounced due to the aging process. SmO_2_ has been shown to be better maintained in 25 y (~73%) than 73 y individuals (~64%) at the same absolute cycling workload (i.e., 50 W) [15]. In this same study, SmO_2_ continued to decrease similarly in both groups as intensity increased to 75 W (young: ~60%; older: ~54%). Interestingly, age-related differences in SmO_2_ are still present as exercise is reported in relative terms. At 50% of maximal workload, younger individuals exhibit a greater SmO_2_ than their older counterparts (young: ~68%; older: ~58%). Disparity in SmO_2_ between groups was maintained as relative exercise intensity increased to 75% of the max (younger: ~63%; older: ~55%). It is important to note that the rate of decrease in SmO_2_ was greater between rest and 50% than between 50 and 75% of the maximal workload for both groups.

There appears to be a muscle-specific effect of exercise on SmO_2_ [16,19], potentially due to differences in fiber type and unequal distribution of blood within muscle [19,48,61]. During submaximal cycling from rest to 120 watts, older adults (~65 y) display lower SmO_2_ in the rectus femoris (RF), biceps femoris (BF), gastrocnemius lateralis (GL), tibialis anterior (TA) and distal portion of the vastus lateralis (VLd) than younger adults (~23 y) [16]. However, SmO_2_ in the proximal end of the vastus lateralis (VLp), vastus medialis (VM) and the gastrocnemius medialis (GM) does not appear to be affected by aging at rest or during submaximal exercise. Variances in SmO_2_ may be attributed to distinctions in actions of specific muscles as well as differences in muscle perfusion, oxygen consumption and dependent upon fiber type composition between different muscles or within the same muscle [16,62]. In support, it has been suggested that there are disparities in the rate of atrophy between muscle fiber types, with type II fibers appearing to be more affected with advancing age [63,64].

The aging process appears to alter VO_2_ kinetics during phase II [18], known as the primary or metabolic phase in which pulmonary VO_2_ rapidly increases until a steady state is met. During phase II, pulmonary VO_2_ closely reflects muscle VO_2_ profiles [17,65,66,67]. This effect was explored by analyzing muscle deoxygenation (Hhb) at the onset of moderate-intensity exercise between older (~68 y) and younger adults (~25 y) with a heavy intensity warm-up (HWU) and without a warm-up (NWU). The key finding from this study related to SmO_2_ demonstrates that a HWU is necessary to prime muscle metabolic processes. The authors report that the time delay before an NIRS-derived increase in deoxygenated Hhb signal was significantly longer following a HWU in the older (HWU: ~34 s vs. NWU: ~22 s), but not the younger group (HWU: ~21 s vs. NWU: ~25 s). The slower response of deoxygenated Hhb signals in older adults following HWU is considered favorable, suggesting that oxygen delivery is increasing at a faster rate than oxygen utilization. In addition, the slowed rate of Hhb signifies improved adjustments in local muscle perfusion and oxygen delivery at the onset of a subsequent moderate-intensity exercise, faster adaptation of VO_2_ kinetics, and decreases the effects of accelerated hypoxia that occurs at the onset of exercise, which are more difficult to adjust to with advancing age.

Similarly, other studies have demonstrated an impact of a HWU prior to submaximal exercise on whole body and local oxygen utilization in older adults (>66 y) [17]. Exercise intensity was set at 80% of each individual’s first ventilatory threshold (VT1). Results indicated the group that completed a HWU before submaximal exercise responded more favorably than the group that did not perform a warm-up. The group that performed a HWU had ~12% reduction in pulmonary and local muscle oxygen deficit during that trial than the group that did not perform a warm-up. Additionally, the rate of adjustment in pulmonary VO_2_ also increased following the HWU trial. This was reflected as the effective time constant (τ’) (~39 s vs. ~36 s) in oxidative metabolism, with the time constant representative of the amount of time it takes for the body’s systems to react to a shift in workload [68]. Furthermore, at VT1, the HWU group exhibited a lower respiratory exchange ratio than the NWU group (NWU: ~0.97 vs. HWU: ~0.91). Collectively, HWU appears to prime metabolic functions for improved oxygen delivery and utilization in aging individuals.

### 3.2. Maximal Exercise

**Figure 2:**
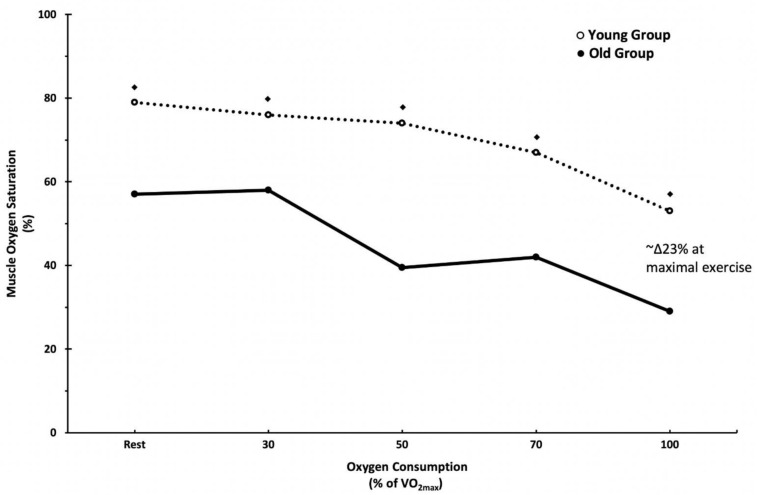
Mean SmO_2_ from rest to maximal exercise in younger (open circle) and older (solid circle) individuals. Statistically significant differences in SmO_2_ were noted at rest and at each point up to VO_2max_ over the course of standard ramp exercise test. ♦ *p* < 0.01 between older and younger groups. Reprinted with permission from ref. [13]. Copyright 1999 Taylor & Francis.

Muscle oxygen saturation has also been shown to decrease at maximal exercise with aging. Study of healthy older (~67 y) and younger (~27 y) individuals demonstrates that aging reduces SmO_2_ by up to ~59% during maximal cycle exercise [13]. While SmO_2_ progressively decreases as a result of increasing exercise intensity in a somewhat similar fashion between younger and older individuals, the older individuals exhibited substantially lower SmO_2_ than their younger counterparts from baseline to maximal exercise (Figure 2). At maximal exercise, SmO_2_ levels were significantly lower in the older compared to the younger individuals (older: ~28% vs. younger ~51%). In support, muscle oxygen saturation at peak exercise has also been reported to be lower in older (~73 y) than younger adults (~25 y) [15]. At peak exercise, Δoxy-Hb/Mb (i.e., an indicator of the balance between oxygen supply and utilization) in the older adults was significantly lower than in the younger population (approximately −6 μmol/L vs. 0 μmol/L). This indicates that the relative concentrations of oxygenated hemoglobin/myoglobin are reduced in the aging population.

### 3.3. Recovery Time

Recent findings have indicated prolonged recovery time following exercise in older individuals. To evaluate this effect, recovery times were compared following a ramp cycle test to exhaustion among healthy older (OA; ~73 y), middle-aged (MA; ~50 y), and younger adults (YA; ~25 y). The authors found a hierarchical recovery pattern following exercise (OA: ~42 s > MA: ~25 s > YA: ~22 s) [15]. These findings demonstrated that older adults had ~51% longer recovery times than middle-aged adults and ~63% longer than younger adults. In contrast, there were no statistical differences in muscle oxygen dynamics during submaximal exercise between MA and YA. Therefore, muscle oxygen metabolism may be preserved in the early stages of aging and progressively exacerbated in the latter stages.

In support of prolonged restoration of SmO_2_ following aerobic exercise, it has been shown that age and exercise training status as well as the combination of both factors impact recovery time [21]. Recovery time among four groups of middle- and older-aged women were analyzed following maximal cycling exercise. Active middle-aged (AM; ~53 y), active older (AO; ~67 y), sedentary middle-aged (SM; ~50 y), and sedentary older (SO; ~66 y) women were evaluated to determine the time required to re-establish 50% SmO_2_ between resting and exhaustion levels (T½ reoxy). Results indicated that a hierarchical pattern in the recovery of SmO_2_ following maximal exercise occurred (SO: 46 s > SM: 36 s > AO: 30 s > AM: 23 s). This corresponds to AO having ~27% slower T½ reoxy time than AM, SO having ~25% slower T½ reoxy time than SM, and SO having ~43% slower T½ reoxy time than AO. These data suggest there is a primary effect of aging on muscle reoxygenation; however, habitual physical activity may slow this effect.

Most studies have assessed SmO_2_ recovery in the lower extremities; however, analysis of SmO_2_ recovery in muscles of the forearm show this region is also affected with advancing age. Forearm muscles were examined in healthy younger (~34 y), healthy older (~67 y), and older adults at risk for CVD (~67 y) to assess the reoxygenation time following handgrip exercise performed at 30% maximal voluntary contraction (MVC) [20]. Handgrip exercises were performed at 60 contractions per minute, 0.5 s contraction/0.5 s relaxation until volitional exhaustion. Muscle oxygen saturation recovery over the first five seconds following exercise termination (SmO_2RR_) was significantly faster in the healthy young group (~1.65 %/s) compared to the healthy older group (~0.92%/s). Furthermore, the older at-risk for CVD group exhibited the slowest SmO_2RR_ (~0.45%/s). The younger group restored oxygen ~57% faster than the healthy older group and ~114% faster than the at-risk for CVD group, respectively. However, no statistical differences were observed between the older groups.

Similarly, analysis of forearm SmO_2_ recovery was performed while utilizing a series of rapid arterial cuff occlusions performed following handgrip exercise at 50% MVC until SmO_2_ decreased by ~50% (~10–30 s), at which point oxygen utilization and mitochondrial function in the flexor digitorum profundus of the forearm were analyzed [12]. After exercise cessation, a series of rapid cuff inflations were employed to generate a muscle oxygen consumption mVO_2_ recovery curve [12]. By measuring SmO_2_ during this protocol and inputting values into a previously established mVO_2_ recovery equation [69,70,71], results indicated that older adults (~72 y) had ~33% longer (~52 s vs. ~37 s) post-exercise mVO_2_ recovery kinetics (i.e., mitochondrial function) compared to their younger counterparts (~25 y).

## 4. Age-Related Changes in the Oxygen Delivery Cascade

**Figure 3:**
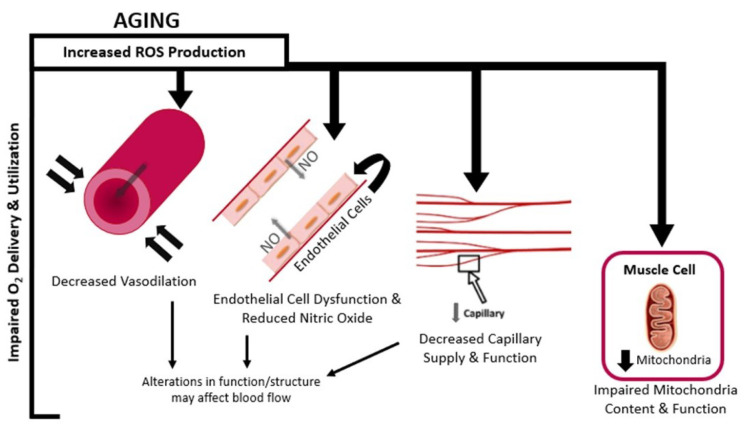
Oxygen delivery to muscle is tightly regulated by blood flow. With advancing age, decreased vasodilation, endothelial cell dysfunction, reduced nitric oxide production, and decreased capillary supply and function are each responsible for reduced blood flow observed with aging and are attributed to mismanagement of ROS. Furthermore, content and function of the cellular machinery tasked with utilizing oxygen, mitochondria, are also impaired with aging. These age-related impairments in the oxygen delivery cascade ultimately lead to reductions in SmO_2_ levels at rest, during exercise, and recovery from exercise.

Oxygen is transported via a series of tightly regulated processes to reach its terminal destination within the myocyte (i.e., mitochondria). Critical steps in the oxygen delivery cascade affected by aging addressed here include blood flow, capillary supply, endothelial cell function, nitric oxide production, and mitochondrial capacity (Figure 3). Due to its ubiquitous nature, ROS is suspected to play a fundamental regulatory role at each point in the oxygen delivery cascade. It is well recognized that ROS is a large contributor to age-related alterations in many physiological systems [72]; however, emphasis is placed here on cardiovascular and skeletal muscle components that are stressed during aerobic exercise and the extent to which excessive ROS interferes with structure and function.

### 4.1. Blood Flow

The initiation of aerobic exercise produces a rapid hemodynamic shift in cardiovascular output to supply adequate blood and, thus, oxygen delivery to skeletal muscle. This increase in oxygen supply is critical to provide cells the resources required to meet metabolic demand. Furthermore, enhanced delivery of oxygen to working tissue is a fundamental component in maintenance of the balance between delivery and utilization (i.e., SmO_2_). Blood flow to skeletal muscle can be increased by as much as 100-fold during intense aerobic exercise [73]. Although, studies have shown that local blood flow is reduced during submaximal [17,18,25,74,75,76,77] and maximal aerobic exercise [78] as a primary effect of aging. Furthermore, older adults (~67 y) have slower [17,18,74] or inadequate redistribution [77] of blood to skeletal muscle in both resting conditions and during aerobic exercise. In addition, arterial vascular conductance has been shown to be ~32% lower along with vascular resistance being ~45% higher in aging [79].

Resting blood flow measurements have been indicated to be ~25% lower in older (~63 y) compared to younger individuals (~28 y). This effect of aging extends into submaximal exercise, as lower extremity blood flow may be as much as ~25% lower in older (~63 y) than younger (~27 y) adults (older: 4.8 L/min vs. younger: 6.2 L/min) [25]. The effect of aging on blood flow is exacerbated during maximal exercise, where blood flow may be as much as ~29% lower in older (~64 y) compared to younger (~22 y) adults (older: 7 L/min vs. younger 9.9 L/min) [78]. As a result of decreased blood flow to skeletal muscle, reductions in oxygen delivery may contribute to lower SmO_2_ levels that are present with aging at baseline measurements [13,15,16], during exercise [13,15], and recovery from exercise [12,15,20,21]. Consequently, limitations in blood flow may create a heightened perception of effort, rapid onset of fatigue, and diminished exercise tolerance [74]. Potential explanations for diminished blood flow during exercise include reduced vascular compliance, altered signaling in vasodilation and/or vasoconstriction, increased peripheral resistance with aging [80], and fewer capillary conduits to perfuse tissue. Diminished blood flow due to reduced vascular conductance is likely a result of prolonged damage to blood vessels; a plausible mechanism for the damage that occurs within these vessels is an imbalance in ROS overproduction, combined with inadequate clearance of ROS [81]. The culmination of ROS-induced damage, within vessels, leads to vascular remodeling [81] and endothelial cell dysfunction [28], further impairing blood flow and oxygen delivery.

### 4.2. Capillary Supply

Due to the complexity of the vascular network, capillarization within skeletal muscle can be quantified in terms of capillary-to-fiber ratio, capillary density, capillary-to-fiber perimeter exchange index (CFPE) and the number of capillaries in contact with each muscle fiber. Studies show that individuals (>60 y) have ~12–25% fewer capillaries in contact with individual muscle fibers than younger adults (~23 y) [26,82,83,84,85]. Given that capillaries directly interface with skeletal muscle, the exposed area of a capillary likely impacts oxygen delivery, and thus cellular uptake [26]. Since oxygen is transported via capillaries to skeletal muscle tissue, diminished capillary supply will impact SmO_2_ levels within the muscle. Therefore, reduced capillarization with aging may be a limiting factor in aerobic performance for older adults. In support, studies have shown that capillarization within skeletal muscle plays a direct role in VO_2max_ in older adults [26,85,86]. CFPE for type I oxidative muscle fibers is strongly correlated to VO_2max_ in both young (~23 y; r = 0.64) and older adults (>60 y; r = 0.88) [85,86]. Capillary contacts (i.e., number of capillaries surrounding a single muscle fiber), as well as the capillary-to-fiber ratio, are ~25% lower in sedentary older men (~65 y) when compared to sedentary young men (~21 y), regardless of fiber type [85]. In support, a 12-year follow-up of older adults was performed (initial testing: ~65 y; 12-year follow-up age: ~78 y) to identify age-related changes in capillary supply in later years of life [87]. Results showed a ~20% reduction in the capillary-to-fiber ratio (initial: ~1.39 vs. follow-up: ~1.08 C:F) over the 12-year period. Interestingly, analysis of aged individuals (~73 y) that maintained regular and structured aerobic exercise training throughout their lifetime demonstrated preserved capillarization (i.e., capillary density, capillary-to-fiber ratio, and capillaries in contact with each fiber (CCEF)) [88].

With advancing age, ROS has been identified as a large contributor to the reduction in capillary supply to muscle, termed capillary rarefaction [89,90]. Reactive oxygen species play an important role as a signaling molecule to help regulate acute vascular responses to metabolic changes such as vasodilation, vasoconstriction, vascular permeability [81], and the formation of new capillaries [91]. However, ROS accumulation may damage microvasculature structures [92]. With aging, the activity of NOX enzymes increases, and consequently, ROS production is upregulated, positively influencing further NOX activity. NOX enzymes are endothelial membrane-bound NADPH oxidases involved in vascular redox signaling responses in the regulation of cell differentiation, proliferation, migration, promotion of capillarization, and vascular tone [81,93,94,95,96,97,98]. Within small vessels, excessive ROS reduces muscle perfusion, while concurrently decreasing vasodilatory response, and increasing micro vessel constriction, which ultimately leads to capillary rarefaction [89].

### 4.3. Endothelial Cells

Endothelial cells (ECs) line the lumen of blood vessels and serve as the biologically active barrier between blood and tissues [99]. These cells are essential in the regulation of blood flow given that they release vasoactive agents that aid in vascular relaxation, vasoconstriction, and tissue perfusion [99]. Endothelial cells also release substances that act upon enzymes imperative to immune function involved in wound healing, angiogenesis, and inflammatory processes [100]. However, ECs are susceptible to age-related biochemical alterations [101]. Endothelial cells, altered with advanced age, are metabolically active, but enter a state of permanent growth repression. This leads to morphological alterations, increasing the sensitivity to pro-apoptotic stimuli, which is primarily a repercussion of attenuated nitric oxide (NO) production [102,103,104]. These changes affect the regulation of biological substances between the bloodstream and the tissues. The accepted methods for evaluation of endothelial cell function include both noninvasive ((i.e., ultrasound flow-mediated dilation (FMD), salbutamol-mediated endothelial function measured by pulse wave analysis (PWA) or pulse contour analysis (PCA), flow-mediated magnetic resonance imaging (MRI), laser Doppler flowmetry, and flow-mediated peripheral artery tonometry/pulse amplitude tonometry (PAT)) and invasive protocols ((i.e., intra-arterial ACh or endothelin infusion and strain-gauge plethysmography or high-resolution ultrasound)) [105].

Flow-mediated dilation has been used to evaluate the progressive decline in endothelial function in both male and female subjects, with the primary goal of assessing rate of blood flow [106]. Results demonstrated that, for men, FMD was generally preserved until ≤40 years of age and, for women, until ~50 years of age. For men, the rate of decline in FMD that occurs beyond 40 y is ~0.21 %/year. Interestingly, though women appear to preserve FMD until a greater age than men (50 vs. 40 y), it appears women experience a greater rate of decline in FMD beyond that point (~0.49 %/year). Supporting that finding, brachial artery dilation was assessed via FMD and was found to be lower in older (~65 y) compared with younger (~29 y) adults (~4.5% vs. ~7.5%) [107]. When assessing brachial artery endothelial function via FMD and PWA in three groups of older adults (60–69 y, 70–79 y and >80 y), it was found that FMD was ~2.9% lower in >80 y when compared to the 60–69 y group, and ~2.7% lower when compared to the 70–79 y group [108]. Additionally, PWA was highest in the oldest group (>80 y: ~1978 cm/s, 70–79 y group: ~1811cm/s, and 60–69 y group: ~1724 cm/s). Using PWA as a method to measure endothelial function provides values for peripheral pressure; higher PWA values indicate there is increased arterial peripheral pressure [109].

One of the primary suspected causes of age-related EC dysfunction is excessive ROS production from surrounding tissues, alongside ROS produced within ECs. Excessive ROS production is problematic as it may lead to the loss of regenerative capacity in ECs [102]. Concurrently, the overproduction of ROS is met with reduced antioxidant defenses in aging [28]. Accumulated ROS within ECs decreases NO, a potent vasodilatory substance produced within ECs through the L-arginine pathway [110]. Exacerbating this interaction, there is the formation of additional ROS, known as peroxynitrite, (i.e., further damage to ECs occurs) [28]. Furthermore, ROS oxidizes tetrahydrobiopterin (BH4), a critical cofactor for endothelial nitric oxide synthase (eNOS) in the enzymatic production of NO [111]. Oxidation of BH4 creates dihydrobiopterin (BH2), which acts as a competitive inhibitor of BH4 [112]. If BH4 is the limiting factor, due to decreases in its synthesis or increases in its oxidation, then eNOS becomes uncoupled leading to the creation of another form of ROS known as superoxide instead of NO [111,112]. Changes to eNOS activity are labeled as “eNOS uncoupling” [113,114,115].

With the goal of modeling advanced age EC physiology, inhibition of NO production, via infusion of N(gamma)-nitro-L-arginine methyl ester (L-NAME), an eNOS inhibitor, can be used to experimentally induce hypertension [116,117]. The first study to implement L-NAME to assess EC function consisted of older adults (~65 y) that performed forearm exercises while a Doppler ultrasound probe simultaneously measured blood flow velocity and vessel diameter. Results indicated forearm blood flow was reduced by ~40% due to eNOS inhibition by L-NAME. The effects of L-NAME simulate age-related ROS-induced damage to homeostatic EC function. Furthermore, those results were compared to a previous study on younger adults (~26 y), using the same testing protocols [118]. It was revealed that the reduction in relative contribution of NO to exercise hyperemia was more pronounced in older (~45%) than in younger adults (~19%). This indicates that older individuals are more susceptible to reduction in NO production, which manifests as decreased exercise hyperemia.

Comparable age-related reductions in vasodilatory responses occurred when similar dose–response protocols were used to assess the degree of EC dysfunction while incorporating strain-gauge plethysmography to monitor changes in skeletal muscle blood flow [119,120,121]. Vasodilatory responses in 119 adults (19–69 y) were assessed for changes that occur over the course of the aging process [120] by administering brachial artery infusions of methacholine chloride, which acts to promote vasodilation by increasing acetylcholine concentration in the synapse [122]. It was found that endothelium-dependent vasodilation decreases steadily over the course of the aging process, despite supra-pharmacologic induced changes in available acetylcholine. These changes are seen as early as the fourth decade of life (30 to 39 y) and are attributed to ROS-related damage to ECs, disrupting the ability of these cells to respond effectively to metabolic changes.

### 4.4. Nitric Oxide

Nitric oxide is produced by almost all cells in the body [123], with the goal to regulate blood pressure and blood flow [124]. Once NO is produced by ECs, it diffuses through the cell to the surrounding smooth muscle tissue promoting vascular relaxation. This will permit dilation of the blood vessels and increase blood flow through the vessels while simultaneously decreasing blood pressure [125]. In general, NO production decreases with age (regardless of health status). This age-related reduction is a product of decreased NO precursors, increased competitive inhibitors of NO, reduced NO synthesis, eNOS uncoupling, and NO scavenging [126].

It has been reported that with aging, there is a steady decrease in endothelial function in older adults (>60 y), with this population experiencing greater than 50% loss of EC function [29,121]. In turn, this loss of function decreases the ability to produce and release NO. Diminished NO production is likely an initial result of ROS-induced damage to ECs, followed by a reduction in NO production due to higher amounts of oxidative stress (i.e., ROS NO scavenging). Impaired NO production throughout the aging process is problematic for a variety of reasons, including the generation of superoxide that can then scavenge NO and create further forms of ROS known as peroxynitrite [28,29]. Peroxynitrite will then continue to deplete NO concentrations within EC cells as well as contribute to endothelial dysfunction [28]. A considerable amount of this degradation occurs from increased ROS scavenging of NO. In addition to this, development of diseases such as hypertension can accelerate deterioration seen in vasculature further impairing EC function and therefore NO production [127]. It has also been found that there is decreased NO production with aging in response to the upregulation of arginase in aged vessels [128,129,130]. Arginase is responsible for breaking down L-arginine used in NO production, further hindering the potential for NO production.

### 4.5. Mitochondrial Function

Skeletal muscle mitochondria play a vital role in regulating cellular processes such as energy production, ROS production/signaling, and apoptosis [131]. Since the discovery of mitochondria in the 1890s, extensive work has been conducted to evaluate their physiological importance; the most well-known function is energy generation in the form of adenosine triphosphate (ATP) [132]. Synthesis of ATP can increase by as much as 100-fold from rest to intense exercise [133,134]. However, age-related reductions in oxygen delivery to mitochondria diminishes mitochondrial function, resulting in reduced ATP production. in vivo evaluation of mitochondrial capacity is generally assessed via phosphorus magnetic resonance spectroscopy (^31^P-MRS) technology, which measures the recovery of creatine phosphate (PCr), or with NIRS technology, which measures the recovery of muscle oxygen consumption (mVO_2_) [48,135].

The reduction in mitochondrial function within muscle presents itself as reduced ability to tolerate prolonged exercise and has been credited as a hallmark sign of the aging process [30,136,137,138,139,140,141,142]. Skeletal muscle of older individuals (~76 y) has ~17% lower in vivo mitochondrial capacity than their younger counterparts (~27 y) during maximal exercise (older: ~0.64 vs. younger: ~0.77 mM ATP/s) [136]. In support of reduced mitochondrial capacity with age, data derived via PCR recovery following exercise utilizing ^31^P-MRS has shown that older adults (~69 y) had ~50% lower oxidative capacity than younger adults (~39 y) (older: ~0.61 vs. younger: ~1.16 mM ATP/s). It was additionally demonstrated that older individuals had reduced mitochondrial volume density compared to the younger group (older: ~2.9% vs. younger: ~3.6%) [137]. Furthermore, there was a significant negative correlation between increased age and decreased mitochondrial volume density and oxidative capacity. Therefore, oxidative capacity per mitochondrial volume was also reduced in the older adults (older: ~0.22 vs. younger: ~0.32 mM ATP/s %). This indicates ATP production relative to mitochondrial volume is diminished with aging.

More recently, NIRS analysis of mitochondrial capacity in three different muscles (GA: gastrocnemius; VL: vastus lateralis; and TA: tibialis anterior) was evaluated between younger (~22 y) and older adults (~68 y) with similar physical activity levels [30]. Mitochondrial capacity was reduced following a 5 s MVC exercise between the age groups for the GA and VL, but not TA, indicating that aging impairs mitochondrial capacity in a muscle-specific manner. Additionally, the VL in the older group had ~25% slower mVO_2_ than the younger group, a likely indication of decreased muscle-specific mitochondrial capacity. This is in agreement with other studies analyzing in vivo PCr recovery in the VL [136,137,138,139,140]. Interestingly, some studies show that mitochondrial content is not different in older (~77 y) and younger (~27 y) adults [136] according to citrate synthase content (younger: ~14.3 vs. older: ~11.5 nmol/min⋅mg protein) and protein content of ETS complexes I–V not being significantly different [136]. However, the P/O ratio was ~21% lower in the older group (younger: ~1.9 vs. older: ~1.5), suggesting reductions in mitochondrial capacity regardless of potentially similar mitochondrial content.

Loss of mitochondrial content and/or function throughout the aging process has been linked to damage incurred from ROS [143,144]. Although, regulated ROS promotes mechanisms for muscle adaptation by stimulating oxidative metabolism, mitochondrial biogenesis, mitochondrial functionality, and antioxidant defense [145]. Mitochondrial homeostasis can be disrupted when the production of ROS occurs in excess of ROS removal [27,146,147], leading to mitochondrial dysfunction and thus mitochondrial autophagy. Data have shown that mitochondrial autophagy is a selective defense mechanism against ROS-induced damage to mitochondrial DNA and electron transport chain function [148,149]. It has been reported that along with the loss of contractile proteins in aging muscle, there is a simultaneous decrease in skeletal muscle mitochondria [136], likely stemming from increased ROS-related mitochondrial autophagy. Skeletal muscle is especially prone to oxidative stress due to the swift biomechanical and biochemical changes in energy requirements and variations in oxygen flux that occur during contractions and changes in metabolic demand. This abruptly alters electron movement and concentrations in the mitochondrial electron transport chain, leading to a higher potential for electron leakage and generation of new ROS [150,151].

## 5. Conclusions

It is generally recognized there is a progressive decrease in VO_2max_ of ~1% per year following age 30 y [8]. More recent developments in NIRS technology have provided the means to assess SmO_2_ as a method to identify age-related changes in local tissue oxygen utilization. These technological advancements have begun to make it clear that aging also reduces SmO_2_ at rest, and during submaximal and maximal exercise, and extends the timeframe for restoration of SmO_2_ following exercise. The age-related reductions that contribute to reduced oxygen delivery and utilization include reductions in blood flow, capillarization and nitric oxide production, and impairments in endothelial cell and mitochondrial function. Compelling data suggest that dysregulated ROS handling ultimately leads to the reductions observed in oxygen delivery and utilization. To expand on our understanding of oxygen delivery and utilization, future research is encouraged to assess the potential effects of sex, mode of exercise, and diet/drug consumption on attenuating consequences associated with aging and reductions in exercise capacity.

## Data Availability

Not applicable.

## Abbreviations

AM—Active middle-aged; ATP—Adenosine triphosphate; ATT—Adipose tissue thickness; BF—Bicep femoris; BH2—Dihydrobiopterin; BH4—Tetrahydrobiopterin; CCEF—Capillaries in contact with individual muscle fibers; CFPE—Capillary-to-fiber perimeter exchange index; CVD—Cardiovascular disease; Cyt_ox_—Cytochrome c oxidase; EC—Endothelial Cell; ENOS—Endothelial Nitric; Oxide Synthase; FMD—Flow-mediated dilation; GA—Gastrocnemius; GL—Gastrocnemius lateralis; GM—Gastrocnemius medialis; ^1^H-MRS—Proton magnetic resonance spectroscopy; Hb—Hemoglobin; Hhb—Deoxyhemoglobin; HWU—Heavy intensity warm-up; L-NAME—N(gamma)-nitro-L-arginine methyl ester; MA—Middle-aged adults; Mb—Myoglobin; MVC—Maximal voluntary contraction; MVO_2_—Recovery of muscle oxygen consumption; NO—Nitric oxide; NIRS—Near-Infrared Resonance Spectroscopy; NIRS_CW_—Near-Infrared Resonance Spectroscopy continuous wave; NIRS_FD_—Near-Infrared Resonance Spectroscopy frequency domain; NIRS_TD_—Near-Infrared Resonance Spectroscopy time domain; NWU—No warm-up; OA—Older adults; O_2_Hb—Oxyhemoglobin; Δoxy-Hb/Mb—Changes in oxyhemoglobin/oxymyoglobin; ^31^P-MRS—31P-magnetic resonance spectroscopy; P/O—Phosphate oxygen ratio; PAT—Flow-mediated peripheral artery tonometry/pulse amplitude tonometry; PCA—Pulse contour analysis; PCR—Creatine phosphate; PWA—Pulse wave analysis; RF—Rectus femoris; ROS—Reactive oxygen species; SM—Sedentary middle-aged; SmO_2_—Muscle Oxygen Saturation; SmO_2RR_—Muscle oxygen saturation over the first five seconds following exercise termination; SO—Sedentary older; TA—Tibialis Anterior; T½ reoxy—50% SmO_2_ between the exhaustion level and resting level; ThB—Total hemoglobin; V_v_(MT,F—Mitochondrial volume density; VLd—Vastus lateralis (distal end); VLp—Vastus lateralis (proximal end); VM—Vastus medialis; VO_2max_—Maximal oxygen consumption; (VT1)—First ventilatory threshold; YA—younger adults.
